# Effect of Psycho-Regulatory Massage Therapy on Pain and Depression in Women with Chronic and/or Somatoform Back Pain: A Randomized Controlled Trial

**DOI:** 10.3390/brainsci10100721

**Published:** 2020-10-12

**Authors:** Sabine B.-E. Baumgart, Anja Baumbach-Kraft, Juergen Lorenz

**Affiliations:** 1 Faculty of Medicine, Institute for Health and Nursing Sciences, Martin Luther University Halle-Wittenberg, 06108 Halle, Germany; 2 M.Sc. Public Health, 24105 Kiel, Germany; baumbach.anja@gmail.com; 3 Department of Biomedical Engineering, Faculty of Life Science, University of Applied Sciences, 21033-Hamburg, Germany; juergen.lorenz@haw-hamburg.de

**Keywords:** massage therapy, chronic back pain, depression, oxytocin, C-tactile fibers, somatoform pain (ICD 10), somatic symptom disorder (DSM-5)

## Abstract

Chronic unspecific back pain (cBP) is often associated with depressive symptoms, negative body perception, and abnormal interoception. Given the general failure of surgery in cBP, treatment guidelines focus on conservative therapies. Neurophysiological evidence indicates that C-tactile fibers associated with the oxytonergic system can be activated by slow superficial stroking of the skin in the back, shoulder, neck, and dorsal limb areas. We hypothesize that, through recruitment of C-tactile fibers, psycho-regulatory massage therapy (PRMT) can reduce pain in patients with cBP. In our study, 66 patients were randomized to PRMT or CMT (classical massage therapy) over a 12-week period and tested by questionnaires regarding pain (HSAL= Hamburger Schmerz Adjektiv Liste; Hamburg Pain adjective list), depression (BDI-II = Beck depression inventory), and disability (ODI = Oswestry Disability Index). In all outcome measures, patients receiving PRMT improved significantly more than did those receiving CMT. The mean values of the HSAL sensory subscale decreased by −51.5% in the PRMT group compared to −6.7% in the CMT group. Depressive symptoms were reduced by −55.69% (PRMT) and −3.1% (CMT), respectively. The results suggest that the superiority of PRMT over CMT may rely on its ability to activate the C-tactile fibers of superficial skin layers, recruiting the oxytonergic system.

## 1. Introduction

Chronic back pain (cBP) has a leading position worldwide in disease-related disability and loss of quality of life [1]. It represents a common health problem, especially among women. According to a GEDA (“Gesundheit in Deutschland Aktuell”, current state of health in Germany) survey from 2009/2010, one in four women reported suffering cBP (lasting >3 months) within the last 12 months [2]. Modern recommendations by national and international health organizations focus on non-drug therapy options in the treatment of chronic (non-specific) back pain. A variety of treatment modalities are suggested, including physical and rehabilitation interventions [3] and instrument-based techniques such as transcutaneous electrical nerve stimulation (TENS) [4], acupuncture [5], low-level laser therapy (LLLT) [6], and shock wave therapy [7]. Additional treatment with pain-relieving medication is recommended. Surgery is not recommended because there is little evidence of its effectiveness [5,8,9]. Chou et al. [10] underlined these recommendations and showed that psychological impairments, e.g., sleep disorders, mood fluctuation, depression, and listlessness, are frequent co-morbidities of chronic pain. A central characteristic of patients with chronic pain is their negative body perception [11], inhibiting cognitive access to therapy [12,13,14]. 

In the 1980s, Groddek and Dogs integrated massage therapy as a form of body therapy into the treatment of chronic pain to gain direct access to the patient’s emotions via their skin and its nervous system, thus facilitating positive body perception and cognitive-behavioral treatment [15,16]. Berg et al. and Listing et al. [17,18] showed how professional therapeutic touch in the form of massage can reduce the physical and psychological symptoms of patients with pain and/or depression. A reduction in pain, mood disorders, and listlessness and fatigue was reported. Furthermore, general psychological tension was reduced, and well-being was increased. Baumgart et al. reviewed randomized controlled trials (RCTs) published from 1996 to 2009 that investigated the effectiveness of massage for patients with depression both as the main diagnosis and a co-morbidity [16]. The authors concluded that the effectiveness of massage therapy depends on the design of the parameters of a) pressure, b) speed, c) direction, and d) rhythm [14,18,19,20,21,22]. Studies on the effects of oxytocin and its interactions with the neuro-physiological system have provided possible explanations for the effect of touch or massage therapy in chronic pain (with and without depression) [23]. In their study, Walker and McGlone illustrated the connection between the type of touch (effect parameters: pressure and time), its neurological transmission of stimuli via C-tactile fibers, and the significance of oxytocin with regard to pain-relieving effects [24]. C-tactile afferent fibers mediate pleasantness of touch and serve a fundamental role in the hedonic function of tactile sensation [25]. Kane and Terrel emphasized the role of touch for child development and propagated the integration of touch into the treatment of developmental trauma [26]. Experimental evidence indicates that activation of C-tactile fibers can significantly alleviate muscle pain [27]. The stimulus of a gentle or moderate touch, transduced in the skin by C-tactile fibers, is transmitted via ascending spinothalamic pathways to the insular cortex, an area of the limbic system. Through connections with the paraventricular nucleus, the thalamus stimulates the synthesis of oxytocin when a touch is perceived as pleasant [23,28,29]. 

Based on these considerations, we hypothesize that pain experience and depressive symptoms can be reduced and physical capacity can be improved by gentle massage that is optimized to activate C-tactile skin afferents. The aim of this study is to examine the effect of both classical and psycho-regulatory massage in patients with cBP on pain experience, depressive symptoms, and physical capacity. 

## 2. Materials and Methods

The study was conducted as a double-blind RCT. The study is registered in the German Registry for Clinical Studies (DRKS00006876), and the protocol was approved by the ethics committee of the University of Halle (Saale), Germany (Nr. 2014-22). 

### 2.1. Eligibility Criteria

The eligibility criteria and baseline data were assessed prior to randomization. The inclusion and exclusion criteria can be found in Table 1 and were defined via extensive literature research. The diagnosis M54 in the ICD-10 (International Statistical Classification of Diseases and Related Health Problems) represents a composite of diagnoses related to back pain. 

Clear diagnosis of chronic pain is difficult, since 90% of the diagnoses do not reveal any apparent clinical findings [30,31]; thus, chronic pain is primarily defined by the duration of the pain [32,33]. Since chronic pain can lead to psychological co-morbidity [34], the diagnostic group of somatoform disorders (ICD-10, F45) was also included. These disorders are generally defined by the occurrence of physical problems without a clear somatic diagnosis.

### 2.2. Participants

**Figure 1:**
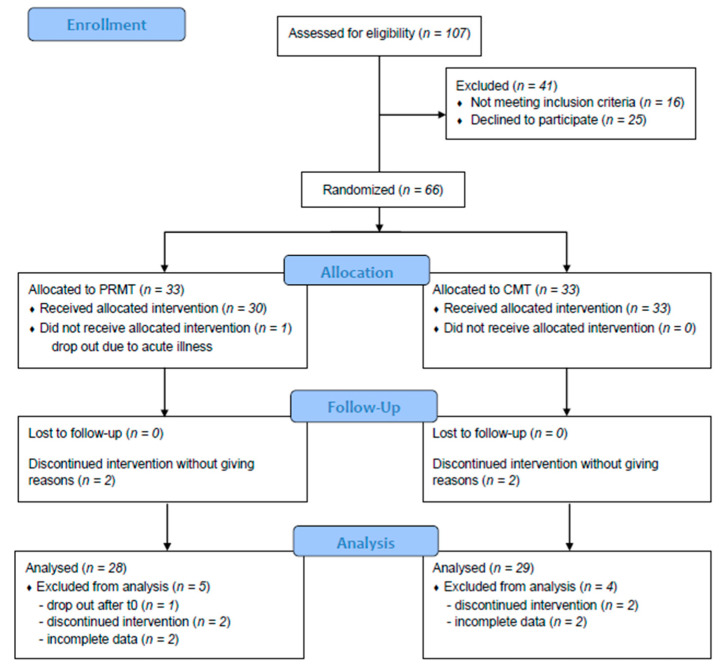
CONSORT (Consolidated Standards of Reporting Trials) flow chart of participants in this study comparing PRMT (psycho-regulatory massage therapy) and CMT (classical massage therapy).

We conducted 107 recruitment interviews. Of these, 41 patients did not participate for the following reasons: *n* = 16 did not meet eligibility criteria, *n* = 3 stated that the number of interventions was too high, *n* = 11 were not comfortable with the nudity required for massage therapy, *n* = 8 found that the questionnaires were too complicated, and *n* = 3 did not want to use massage oil. Overall, *n* = 66 patients were randomized into either the intervention or control group using hidden lots covered in envelopes. One patient was excluded prior to the first treatment due to acute illness; 61 patients completed the treatment. Two patients in each treatment group discontinued the treatments without giving any reason (see Figure 1).

### 2.3. Interventions

Interventions took place in the physiotherapy practice operated by the principle investigator (SB). The outpatient setting was chosen to maximize external validity. The intervention group received psycho-regulatory massage therapy (PRMT) and the control group received classical massage therapy (CMT). Patients were blinded towards the type of massage they received. However, they were aware of the study’s aim to compare the two types of massage. Data analysis was blind towards a patient’s treatment method. All therapists employed in the practice participated in the study (*n* = 7). The design of effect parameters differed between the intervention and control groups in terms of pressure, speed, direction, and rhythm [12,14], as shown in Table 2. The interventions also differed in respect to the target organ and body areas treated. PRMT targets the skin and the superficial fascia, whereas CMT targets all layers of the tissue, including the periosteum. The PRMT unfolds from three partial massages to a full body massage. It is applied with warm oil in both supine and prone body positions and involves soft to moderate intensities of continuous slow strokes. They are uninterrupted throughout the entire session except during the change from supine to prone body position. The therapist does not touch the different body parts in separate sequences, but moves in harmonious transitions from limbs to trunk to create a whole-body experience. An extended description of the PRMT technique is added as supplemental material. In contrast, CMT is applied to the back alone and extends from the sacrum to the neck. 

The intervention group was treated by seven therapists who received professional training to standardize the performance of PRMT, which was applied for 30 to 60 min [35,36]. The control group received 20 min of CMT, which was not standardized but applied individually, reflecting standard care within the German statutory health insurance scheme. All treatments were applied non-verbally in a closed therapy room with only the patient and therapist present. Patients were treated by the same therapist during the whole study period, for optimal therapeutic effectiveness [18,21]. Each group received 10 treatments overall, which were scheduled twice a week. 

### 2.4. Data collection

**Figure 2:**
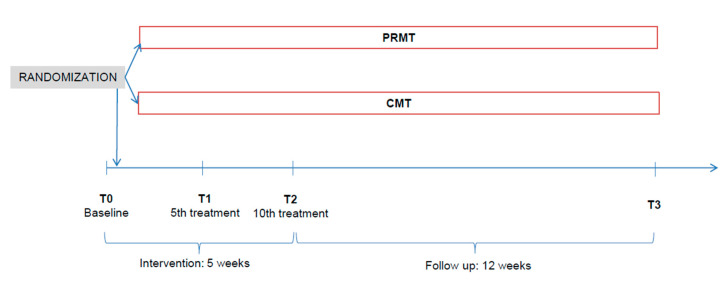
Study timeline (T, time of measurement; PRMT, psychoregulatory massage therapy; CMT, classical massage therapy).

After 3 months, follow-up data were collected. Figure 2 shows the structure and course of the study. Data were collected via questionnaires, handed out by the therapists and filled by the patients themselves. Baseline data (T0) were collected prior to randomization and the first intervention. At T1 (5th treatment), T2 (10th treatment), and at follow-up (T3), data were collected after the interventions. 

Pain was assessed using the Hamburg Pain Adjective List (Hamburger Schmerz Adjektiv Liste, HSAL), a multi-dimensional questionnaire for pain experience in adults with acute or chronic pain. The HSAL consists of 37 adjectives, 21 of those describing the affective experience of pain (pain suffering + pain anxiety) and 16 describing the sensory experience of pain (pain rhythm + pain acuity). Each item can be answered on a scale from 0 (not correct at all) to 6 (completely correct), and the answers are added up to create a total score (maximum of 222, corresponding to a maximum of pain) [37]. The validity of the HSAL has been assessed by several studies in different clinical situations (Cronbach’s alpha of the primary scales is between 0.80 and 0.90) [38]. The HSAL questionnaire is especially suitable in connection with psychiatric scales (depression, anxiety) and also has good applicability for monitoring patient health. 

Depressiveness was measured using the Beck Depression Inventory (BDI-II), scaling 0–3 for each item; the maximum overall score is 63 points [39,40]. The severity of depression is categorized into five groups:

0–8 no depression;
9–13 minimal depression;
14–19 mild depression;
20–28 moderate depression;
29–63 severe depression [41].

The clinical relevance of the respective changes in depression was assessed according to the criteria of Hiroe et al. [42]. Accordingly, a 5-point change in BDI-II score indicates minimal relevance, 10–19 points is moderate, and more than 20 points corresponds to a strong effect.

Quality of life was measured using the Oswestry Disability Index (ODI), a self-rating questionnaire about disability in patients with back pain [43]. The questionnaire consists of 10 items, which are categorized into (1) physical complaints (or disability), (2) activity, and (3) participation according to the International Classification of Functioning, Disability and Health (ICF). A maximum score of 50 indicates maximum impairment. The scores are then converted into percentages depending on the number of questions answered. Mannion et al. [44,45] and Hooff et al. [46] investigated the validity of the German version. Their results showed that the ODI is a good measurement tool for assessing a patient’s disability due to back pain (Cronbach’s alpha = 0.90). The total ODI score represents a percentage and is interpreted as follows:

0–2 minimal disability (all activities of daily living (ADL) are mostly possible, often no therapy necessary, activation of life is enough);
21–40 moderate disability (participation is already limited and incapacity to work often occurs, conservative treatment);
41–60 severe disability (pain is the main problem and ADL are affected, intensive diagnostics are necessary);
61–80 crippled (all areas of life are affected);
81–100 bedridden or the patient exaggerates [47,48].

### 2.5. Sample Size and Randomization

For sample size calculation on the basis of overall pain experience as the primary outcome, alpha was set to 5%, statistical power should be at least 90% (SD 2.0), and the drop-out rate was expected to be 20%, resulting in a group size of *n* = 33. Block randomization was carried out with a block size of 10 by an independent statistician from University of Halle, Saale (Germany).

### 2.6. Statistical Analysis

Given the ordinal data type of all questionnaires (HSAL-total, HSAL-affective, HSAL-sensory, BDI-II-score, and ODI (%)) that were, in at least one session, not normally distributed, as tested with the Kolmogorov–Smirnov test, we applied the Wilcoxon rank-sum test for two independent samples to analyze group differences (CMT vs. PRMT) at baseline (T0) and to test the magnitude of change from baseline to the T1, T2, and T3 time points (SPSS, Version 26). A significant difference (two-tailed test) was accepted at *p*-values below 0.05. To test the effect of sessions, we applied the Friedmann ANOVA test separately for CMT and PRMT. In case of significance, we used the Wilcoxon test for paired samples. According to the total of six comparisons, we used Bonferroni correction, resulting in a *p*-value of 0.008 as the threshold for significance.

## 3. Results

The results of the non-parametric comparison using the Wilcoxon rank-sum test for two independent samples are summarized in Table 3 for all study parameters. The baseline condition (T0) yielded non-significant differences for all questionnaire scores.

HSAL total, affective, and sensory scores for pain: The total scores and the affective and sensory subscores of HSAL were significantly smaller in the T2 and T3 sessions in the PRMT group compared to the CMT group. No difference appeared in session T1. The Friedmann ANOVA yielded a non-significant effect of treatment session for CMT in HSAL-Total (*p* = 0.56), weak significance for HSAL-Affective that failed to reach the significance criterion in any of the six comparisons, and an absence of significance for HSAL-Sensory (*p* = 0.127). In contrast, all HSAL parameters were statistically significant for the effect of treatment session in PRMT (*p* < 0.001). Post hoc Wilcoxon testing revealed significant decreases in all HSAL parameters for comparisons T3 vs. T0, T4 vs. T0, T3 vs. T2, and T4 vs. T2 (*p* < 0.001; Bonferroni-corrected).

BDI-II score for depression: PRMT reduced the intensity of depression more than CMT already during T1, but more strongly during sessions T2 and T3. The Friedman ANOVA resulted in a significant session effect in CMT (*p* = 0.03); however, no post hoc comparison reached the Bonferroni-corrected threshold criterion. In contrast, PRMT had a significant effect of session with statistically significant post hoc Wilcoxon testing in all six comparisons (*p* < 0.001): T0 vs. T1, T0 vs. T2, T0 vs. T3, T1 vs. T2, T1 vs. T3, and T2 vs. T3. Thus, the BDI-II score continuously decreased over the three months of treatment. Overall, the severity of depression decreased by 55.69% with PRMT, from a moderate to minimal level of severity on average [45]. Under CMT, the mean BDI-II score changed by −3.1% over the whole study period. 

ODI (%) score for disability: The progression of improvement in functional status achieved by PRMT in comparison to CMT is quite similar to that for depression. A weak, yet significantly better improvement in PRMT than in CMT occurred in T1, but much greater treatment differences appeared in T2 and T3. The Friedmann ANOVA showed non-significant change by CMT (*p* = 0.56). In contrast, there was a significant change in ODI (%) by PRMT (*p* < 0.001) with significant post hoc differences in all comparisons, except for T3 vs. T2. Table 4 shows the distribution of patients within the different levels of disability (reflecting limitations in quality of life, daily activities, and participation) at baseline and follow-up measurements. Before intervention, the vast majority (90.62%) of the PRMT group had a moderate to severe level of disability. At follow-up, 86.66% of the patients had minimal to moderate levels of disability. On average, the degree of disability improved by at least one level in 90% of the PRMT group, while CMT did not lead to any substantial change. The effect size according to Cohen, ƒ = 0.47, corresponds to a strong effect. 

## 4. Discussion

The primary aim of this study was to examine the effect of two forms of massage therapy in patients with chronic back pain (cBP). According to the general consensus about pain as a multidimensional experience [49,50] pain was assessed via HSAL (the Hamburg Pain Adjective List). In the group receiving PRMT, the decrease in the HSAL total score was statistically significant (−57.7 points), relating to an improvement of 46%. In contrast, the HSAL total score improved only by 5.6% in the group receiving CMT, failing to reach statistical significance and indicating no clinical relevance. In terms of decrease in symptoms of affective and sensory pain experience, PRMT thus shows strong superiority over CMT. Consistent with our results, Cherkin et al. [51] investigated 10 massage applications with a duration of 50–60 min and compared “structural” massages with relaxing massages. Relaxing massages were superior to structural massages, the latter being comparable to CMT. The follow-up at 10 weeks showed that the results persisted in both groups; thus, a long-term effect analogous to the results of the present study could also be observed. Without emphasizing different techniques, Wallach et al. [52] observed a sustained long-term effect of CMT after three months involving 10 applications of massage therapy of 20 min each over 10 weeks. Hamre et al. [53] investigated the effect of rhythmic massage therapy in a four-year prospective cohort study. They examined 85 chronically ill patients, 45 of whom suffered from chronic back or neck pain. Eighteen of the patients also suffered from depression and fatigue. The disease and symptom scores used in that study, each on a scale of 0 to 10, improved significantly. The disease score decreased from 6.3 to 2.77 points, and the symptom score decreased from 5.76 to 3.13 points. The SF-36 questionnaire also showed an improvement in the physical component score and the mental score. Thus, the symptoms of chronic disease were reduced, and at the same time, the quality of life of the patients improved.

Models from neuro-biological research might explain the positive effects of massage and the role of their specific techniques. Studies using microneurography have indicated the existence of low-threshold slowly conducting C-fibers in superficial skin layers that are predominantly activated by soft and low-velocity (3 cm/s) stroking stimuli [27]. In contrast, stronger pressure stimuli penetrating into deeper subepidermal skin layers activate rapidly conducting A-tactile afferents. A-tactile and C-tactile afferents are regarded as important for discriminant and affective dimensions of touch perception, respectively [28]. Accordingly, A-tactile fibers are predominantly abundant in the glabrous skin of the hands, whereas C-tactile fibers predominate in the hairy skin of the limbs and back. Several authors have pointed to the importance of the type of touch to be applied: slow, harmonic, and rhythmic with moderate pressure [24,54,55,56,57]. 

Boehme et al. compared patients suffering from fibromyalgia with healthy controls by using functional magnetic resonance imaging (fMRI) imaging of brain activity in response to selective C-tactile stimuli and additionally analyzed the voxel-based morphometry in areas of the limbic cortex [58]. They observed an abnormal pattern of deactivation and activation within the posterior insula during pleasantness and pain ratings, respectively, and a reduced grey matter density in the hippocampus and anterior insula. The authors interpreted their results as indications of anhedonia to gentle touch in fibromyalgia. Notably, classical massage is reported to worsen pain, whereas soft skin stroking, lymphatic drainage, and superficial vacuum massage alleviate pain in fibromyalgia patients [59]. Liljencrantz and Olausson [56] also reported anxiety-reducing effects of stimulating C-tactile afferents. 

Craig [60] and Devue et al. [61] identified a functional network between the anterior insula and the cingulate gyrus, which serves primarily for self-recognition and awareness of one‘s own body. External impulses like touch and their subjective processing are thus integrated into emotional experiences. Older studies identified interoceptive afferents of primates as correlates of a so-called “gut feeling”, which represents a complex integration of sensory perceptions and corresponding emotional responses. In the early 1990s, Damasio demonstrated that emotional and mental responses to stimuli mediate spino-thalamic and insular impulses that are integrated into body perception [62]. Paulsen and Stein reported that patients with depression and/or anxiety disorders experience significantly altered interoceptive signal processing. Signals are passed on blurred and amplified, so that homeostatic states are difficult to predict [63].

Several studies have shown an interaction of C-tactile afferents with the oxytonergic system in reducing pain and improving body perception [24,54,55]. Pfeifer et al. [64] and Uvnäs-Moberg and Peterson [23] postulated a positive influence on pain memory via the limbic system and activation of the oxytonergic system. This hypothesis was confirmed by follow-up data. A long-term effect was shown in both groups at three months post-intervention. This is coherent with the neuro-biologic findings by Lund et al. [65], who reported a long-term pain-reducing effect of soft massage-like touch through interaction of the oxytonergic system with the opioid system and activation of periaqueductal grey neurons. Miranda-Cardenaz et al. [66] and DeLaTorre [67] detected oxytocin receptors in the laminae of the dorsal horns. By micro-stimulation of the paraventricular nucleus and intrathecal administration of oxytocin, they were able to demonstrate an inhibited stimulus response of the wide dynamic-range neurons in the spinal horn in chronic pain syndrome. The authors considered this to be a descending oxytonergic control mechanism that influences chronic pain perception. Oxytocin also plays an antagonistic role in the glutamatergic spinal sensory conduction of acute pain stimuli [68]. Oxytocin inhibits the conduction of pain in the protopathic ascending pathways and plays a role in the function of the opioid system and gate control mechanism [64,65]. Thus, oxytocin can inhibit acute and chronic pain stimuli at different levels of the central nervous system (CNS).

The results on depression severity in our study, as measured by the BDI-II questionnaire, lend further strong support for the importance of C-tactile stimulation underlying the superior efficacy of PRMT over CMT. In the PRMT group, improvements on the BDI-II correspond to a moderate clinical effect [46]. In contrast, the severity of depression did not change significantly following CMT. These results support findings from a systematic review by Baumgart et al. that found massage therapy to be effective for depression and anxiety as a primary diagnosis and a co-morbidity, respectively [16]. Finally, disability and activity improved significantly within the PRMT group. Inter-group analysis also showed a significant effect in favor of PRMT compared to CMT. The PRMT group showed an improvement rate of 37.76%—the CMT group, only 3.46%. Changes of at least 18% are considered clinically relevant [46]. In this study, an important aim of chronic pain treatment (increase in function and activity) was achieved with PRMT [61]. Positive psycho-emotional effects, including a decrease in depressive symptoms, increase the motivation to maintain physical integrity, which is associated with an increase in personal activity [20,69,70]. Perceiving the back as a pleasant body part might reduce negative self-referential processing and open the patients towards treatments aiming at cognitive emotion regulation, e.g., mindfulness training [71]. 

There are limitations of our study. Since this study was conducted in the outpatient setting of a physiotherapy practice, the level of treatment standardization was low. Factors such as the patient’s daily routine; sleep, waking, and eating rhythm; and time of intervention were not standardized to increase external validity. Treatment prescription was done by private orthopedic surgeons and general practitioners using the ICD-10 codes M54 and F45, which qualified the patients for study inclusion. There might have been variability among doctors in the use of these diagnoses. Yet, no patient had indication of specific back diseases causing their pain. Use of the term “somatoform” might be debatable, and it differs between the DSM (Diagnostic and Statistical Manual of Mental Disorders) and ICD classifications. During the update to DSM-5, the class of “somatoform disorders” was changed to “somatic symptom disorder”, whereas the term is still used in the ICD-10. Furthermore, patients were allowed to use their pain medication as usual and receive other therapies, introducing a certain risk of bias to our results. Future studies could examine how the use of analgesics can be reduced by PRMT. Standard regulations of health care insurances allow 20 to 30 min of treatment duration for CMT, but 60 min for PRMT, because the latter involves a whole-body massage. Thus, longer treatment sessions might have contributed to the superiority of PRMT over CMT. The follow-up period of our study was three months, to increase comparability with other studies [16]. However, future studies could choose a longer follow-up period to better assess the long-term effects of PRMT intervention. Although the BDI-II score yielded no significant group differences at baseline, there was no stratification of patients according to the severity of depressive symptoms by an expert in psychiatry. Our results strongly suggest that patients with indications of depressive comorbidity benefit the most from PRMT. Although our a priori concept and conduct of PRMT aimed at an optimization of recruiting C-tactile afferent activity, we have no physiological proof of its importance in our study. Heart rate variability analysis has been found to be sensitive to the pleasantness of C-tactile stimulation [72] and appears to be compatible with study designs such as the one presented here.

## 5. Conclusions

Our results indicate that psycho-regulatory massage therapy (PRMT) is more effective than classical massage therapy (CMT) in reducing pain and depression and enhancing physical capacity and activity in patients suffering from chronic unspecific back pain. Unlike CMT, PRMT characterizes a massage technique by which the therapist applies soft and slow strokes upon the skin of the back, neck, shoulders, and upper arms that specifically activate C-tactile fibers. Future studies should examine the importance of individual differences of co-morbidity with depression in patients suffering from chronic unspecific back pain for the superiority of PRMT over CMT. This knowledge would improve the selection of individualized physical therapy options for these patients.

## Figures and Tables

**Table 1:** List of eligibility criteria for study participation.

Inclusion Criteria	Exclusion Criteria
Back pain diagnosis according to the ICD-10 code: M54 and F45, medically certified Pain duration of >6 months Patient age 18–75 years Voluntary participation in the study Sufficient knowledge of German to understand the questionnaires	Limited ability for consent to study participation Inflammatory disorders Open wounds Ongoing application for pension

**Table 2:** Effect parameters of the interventions.

Parameters	PRMT	CMT
Pressure	soft to moderate	soft to strong
Speed	continuously decreasing to a speed of 10–3 cm per second	a slow speed is recommended for pain reduction not clearly defined, depends on training and individual therapist
Direction	Body areas are connected ending with cranial to caudal strokes	depends on therapist’s training from the origin to the beginning of a muscle
Rhythm	harmonious and constant contact with the patients until the end of the treatment	not defined

PRMT, psyochregulatory massage therapy; CMT, classical massage therapy.

**Table 3:** Results (mean and SD) for pain experience (HSAL total score and affective and sensory subscales), depressive symptoms (BDI-II), and disability (ODI); results of the Wilcoxon rank-sum test for independent samples tested for effect of treatment (PRMT vs. CMT) and *p*-values for two-tailed comparisons.

		T0	T1	T2	T3
	CMT	108.72	113.34	104.76	102.62
	SD	51.86	61.75	62	61.92
HSAL-Total	PRMT	124.82	115.04	72.14	67.32
	SD	57.81	51.38	36.16	32.86
	Wilcoxon_Z	−1.76	−1.04	−4.47	−3.65
	*p*	n.s.	n.s.	<0.001	<0.001
	CMT	66.38	67.03	61.79	60.86
	SD	34.79	38.7	38.28	38.49
HSAL-Affective	PRMT	75.86	70.39	44.75	43.93
	SD	33.13	30.68	23.3	21.83
	Wilcoxon Z	−0.84	−1.763	−3.54	−3.38
	*p*	n.s.	n.s.	<0.001	<0.001
	CMT	44.66	46.31	42.97	41.69
HSAL-Sensory	SD	23.73	26.34	26.41	26.45
	PRMT	48.93	44.29	27.39	23.75
	SD	26.91	23.72	16.17	15.8
	Wilcoxon Z	−0.61	−1.91	−3.86	−3.94
	*p*	n.s.	n.s.	<0.001	<0.001
BDI-II score	CMT	19.3	20.52	19.59	18.69
	SD	9.64	10.61	11.4	10.36
	PRMT	23.29	20.46	12.25	10.32
	SD	11.58	10.7	7.62	5.9
	Wilcoxon Z	−0.6	−2.35	−4.21	−4.41
	*p*	n.s.	<0.05	<0.001	0.001
ODI (%)	CMT	39.9	39.73	39.09	38.52
	SD	14.1	16.23	16.86	16.42
	PRMT	38.26	36.12	25.95	23.82
	SD	12.5	14.06	10.49	11.6
	Wilcoxon Z	−0.09	−2.09	−4.19	−4.2
	*p*	n.s.	<0.05	<0.001	<0.001

BDI-II: Beck Depression Inventory, 2nd version; ODI: Oswestry Disability Index; PRMT: Psycho-regulatory massage therapy; CMT: classical massage therapy; n.s.: not significant.

**Table 4:** Distribution of patients at baseline (T0) and follow-up (T3) according to the respective levels of disability.

Level of Disability	PRMT *n* = 28		CMT *n* = 29	
	T0	T3	T0	T3
Minimal	6.25%	43.33%	9.09%	10.34%
Moderate	46.87%	43.33%	45.45%	51.73%
Severe	43.75%	13.34%	33.34%	20.69%
Crippled	3.12%	0%	12.12%	17.24%
Bedridden	0%	0%	0%	0%
